# Runx3 Regulates CD8^+^ T Cell Local Expansion and CD43 Glycosylation in Mice by H1N1 Influenza A Virus Infection

**DOI:** 10.3390/ijms25084220

**Published:** 2024-04-11

**Authors:** Qin Hao, Suman Kundu, Sreerama Shetty, Hua Tang

**Affiliations:** Department of Cellular and Molecular Biology, The University of Texas Health Science Center at Tyler, Tyler, TX 75708, USA; qin.hao@uthct.edu (Q.H.); skundu1@uthsc.edu (S.K.); sreerama.shetty@uthct.edu (S.S.)

**Keywords:** Runx3, influenza A virus, lung, CD8^+^ T cells, CD43 glycosylation

## Abstract

We have recently reported that transcription factor Runx3 is required for pulmonary generation of CD8^+^ cytotoxic T lymphocytes (CTLs) that play a crucial role in the clearance of influenza A virus (IAV). To understand the underlying mechanisms, we determined the effects of *Runx3* knockout (KO) on CD8^+^ T cell local expansion and phenotypes using an inducible general *Runx3* KO mouse model. We found that in contrast to the lungs, *Runx3* general KO promoted enlargement of lung-draining mediastinal lymph node (mLN) and enhanced CD8^+^ and CD4^+^ T cell expansion during H1N1 IAV infection. We further found that *Runx3* deficiency greatly inhibited core 2 O-glycosylation of selectin ligand CD43 on activated CD8^+^ T cells but minimally affected the cell surface expression of CD43, activation markers (CD44 and CD69) and cell adhesion molecules (CD11a and CD54). *Runx3* KO had a minor effect on lung effector CD8^+^ T cell death by IAV infection. Our findings indicate that Runx3 differently regulates CD8^+^ T cell expansion in mLNs and lungs by H1N1 IAV infection. Runx3 is required for CD43 core 2 O-glycosylation on activated CD8^+^ T cells, and the involved Runx3 signal pathway may mediate CD8^+^ T cell phenotype for pulmonary generation of CTLs.

## 1. Introduction

Influenza is a contagious acute respiratory disease that can trigger exacerbations of airway and lung disorders as well as cardiovascular diseases [1,2,3]. Influenza infection causes considerable morbidity and mortality and affects people at all ages, hence posing a threat to human health globally [2,3]. Influenza A virus (IAV) infection elicits host innate and adaptive immune responses that are the key host antiviral defense mechanisms to restrain virus replication and spread [4,5,6]. It has been demonstrated that pulmonary CD8^+^ cytotoxic T lymphocytes (CTLs) play a central role in the clearance of IAV due to their ability to specifically recognize and directly destroy the IAV-infected cells [6,7,8]. Those CTLs also can recognize internal and conserved IAV epitopes, offering cross-reactive protection against different IAV variants and subtypes [9,10,11,12].

In response to IAV infection, naive CD8^+^ T cells in lung-draining mediastinal lymph node (mLN) are primed by lung conventional dendritic cell (DC) subset-1, become activated, and undergo reprogramming to express a group of new gene products, which facilitate their local expansion, differentiation, and infiltration into inflamed lungs to provide antigen-specific protective immunity [6,7,8,13,14]. Trafficking of CD8^+^ T cells during viral infection is a highly dynamic process that is mediated by a variety of receptor–ligand interactions. Extravasation of activated CD8^+^ T cells from the circulation to non-lymphoid tissues is initially mediated by the capture of activated T cells with P- and E-selectins on inflamed vascular endothelium [14,15,16,17,18]. Endothelial P- and E-selectin proteins can bind to the specific oligosaccharide structures on T cell surface proteins called P- and E-selectin ligands that include P-selectin glycoprotein ligand-1 (PSGL-1), CD43, E-selectin ligand-1 (ESL-1), and CD44 [14,15,16,17,18]. P- and E-selectin ligands only become functional when they undergo core 2 O-linked glycosylation found on activated T cells. Naïve T cells synthesize core 1 but not core 2 O-glycans and are unable to bind P- and E-selectins and hence are essentially excluded from entering non-lymphoid tissues. Thus, core 2 O-glycosylation is essential for the generation of functional P- and E-selectin ligands and plays an indispensable role in homing of activated CD8^+^ T cells to non-lymphoid tissues including the lungs in vivo [14,15,16,17,18,19,20,21,22]. In contrast to T cells, innate immune cells such as neutrophils and monocytes often express functional P- and E-selectin ligands constitutively [19]. Moreover, core 2 O-glycosylation of CD43 has recently been identified as a sensitive marker of Notch signaling in activated CD8^+^ and CD4^+^ T Cells [23]. Notch signaling has emerged as a critical regulator of CD4^+^ and CD8^+^ T cell activation, differentiation, and effector function [24]. It has been shown that impaired Notch signals in T cells reduce their protective functions against bacterial, viral, fungal, and parasitic infections in mice [25,26,27,28].

Transcription factor Runx3 is required for embryonic development and, together with Runx1, mediates thymopoiesis [29]. A general *Runx3* knockout (KO) in C57BL/6 and BALB/c mice leads to animal death soon after birth [30,31]. We recently generated an inducible *Runx3* general KO mouse model and showed that *Runx3* KO profoundly reduced the numbers of pulmonary CD8^+^ CTLs during IAV infection, while it modestly increased pulmonary CD4^+^ T cell populations [32]. To understand Runx3 regulation of pulmonary CD8^+^ CTLs due to IAV infection, we aimed to unravel the effects of *Runx3* KO on CD8^+^ T cell populations, their CD43 glycosylation, and surface expression of activation markers and cell adhesion molecules in IAV-infected mLNs and lungs. Our findings indicate that Runx3 is required for core 2 O-glycosylation of selectin ligand CD43 on activated CD8^+^ T cells and differently regulates CD8^+^ T cell expansion in mLNs and lungs in response to H1N1 IAV infection.

## 2. Results

### 2.1. A General Runx3 KO in Adult Mice Promotes mLN Enlargement and Expansion of CD8^+^ and CD4^+^ T Cells following H1N1 IAV Infection

**Figure 1:**
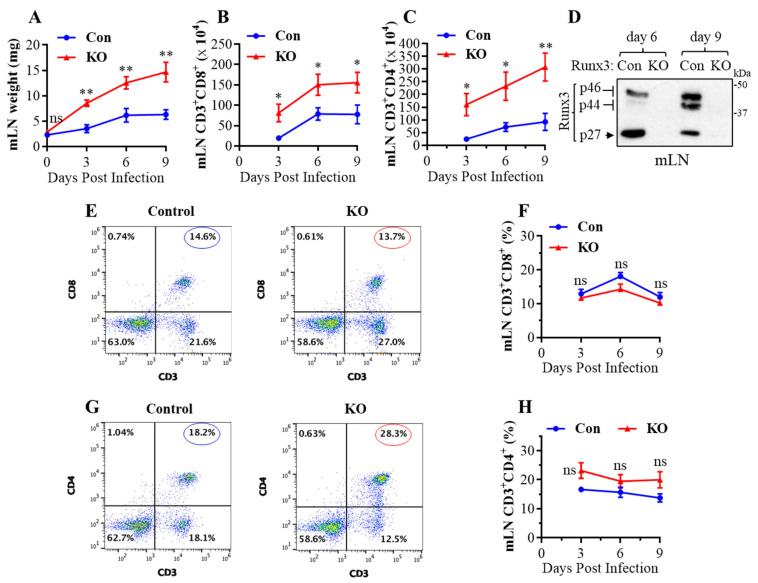
A general *Runx3* KO in adult mice promotes mLN enlargement and expansion of CD8^+^ and CD4^+^ T cells in response to H1N1 IAV infection. Age- and sex-matched littermate control (Con) and *Runx3* general KO (KO) mice were infected intranasally with 1 LD_50_ of H1N1 PR8 strain for 9 days. (**A**) Increased weight of mLNs by *Runx3* KO during IAV infection. Results are expressed as means ± SE. Day 0, n = 7; day 3 post IAV infection (pi), n = 3; days 6 and 9 pi, n = 6. (**B**,**C**) KO of *Runx3* enhances mLN expansion of CD8^+^ and CD4^+^ T cells during IAV infection. CD8^+^ (**B**) and CD4^+^ (**C**) T cells in mLNs were identified by the selected surface markers, and their absolute cell numbers are shown in the bar graphs. Results are expressed as means ± SE. Day 3 pi, n = 3; days 6 and 9 pi, n = 6. (**D**) Verification of *Runx*3 KO in mLNs by Western blot analysis. Results represent the findings of three independent experiments. (**E**–**H**) KO of *Runx3* has a minor effect on the frequency of CD8^+^ and CD4^+^ T cells in mLN during IAV infection. Representative FACS plots evaluated on day 3 pi for CD8^+^ and CD4^+^ T cells are shown in (**E**,**G**), respectively. The cell frequencies of CD8^+^ (**F**) and CD4^+^ T cells (**H**) are shown in the line graphs. Results are expressed as means ± SE. Day 3 pi, n = 3; days 6 and 9 pi, n = 6. Two-way ANOVA followed by Bonferroni’s correction and an unpaired Student’s *t* test were employed for comparison in (**A**–**C**,**F**,**H**) * *p* < 0.05; ** *p* < 0.01 vs. control mice. ns, not significant.

Runx3 is essential for normal embryonic development, and a general *Runx3* KO leads to animal death soon after birth [30,31]. To understand the role of Runx3 in adult mice, we recently developed an inducible general *Runx3* KO mouse model and reported that *Runx3* KO minimally affected thymic function and animal survival in adult C57BL/6 mice under normal conditions [32]. Both control and *Runx3* KO mice also had a small mLN with comparable weight under the steady state condition (day 0) (Figure 1A). Following IAV infection, respiratory conventional DCs migrated to the lung-draining mLN and presented the processed viral antigens to cognate T cells to initiate naïve T cell activation, proliferation, and differentiation [6,7]. Interestingly, we found that a global KO of *Runx3* markedly augmented mLN enlargement and weight during H1N1 IAV infection (Figure 1A). This was correlated with a marked and significant increase in the absolute CD8^+^ (Figure 1B) and CD4^+^ (Figure 1C) T cell populations in mLN by *Runx3* KO during IAV infection. KO of *Runx3* in mLNs was verified by Western blot analysis as three Runx3 isoform bands (p46, p44 and p27) [33] were virtually not detected in mLN homogenates from *Runx3* KO mice (Figure 1D). In addition, KO of *Runx3* did not significantly affect the frequency of CD8^+^ (Figure 1E,F) and CD4^+^ (Figure 1G,H) T cells in mLN during IAV infection. These data indicate that a general *Runx3* KO promotes mLN enlargement and expansion of activated CD4^+^ and CD8^+^ T cells in response to H1N1 IAV infection.

### 2.2. Runx3 KO Inhibits Core 2 O-Glycosylation of Selectin Ligand CD43 on Activated CD8^+^ T Cells in Response to H1N1 IAV Infection

**Figure 2:**
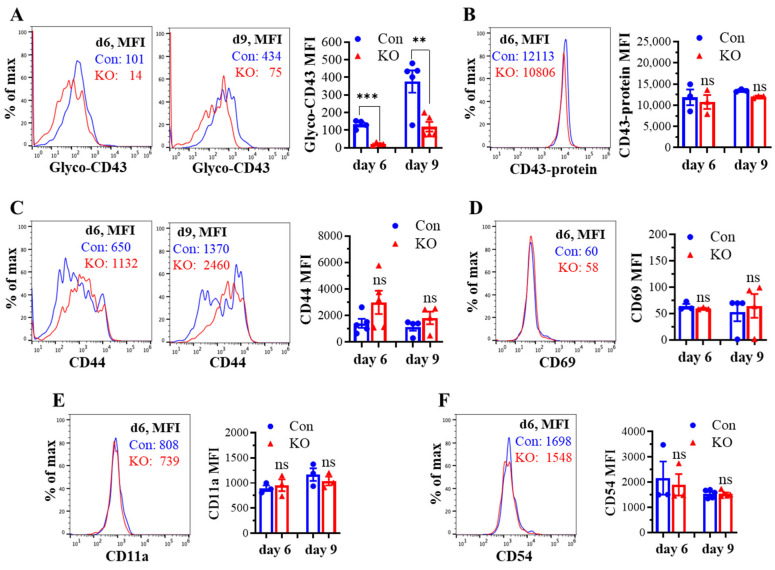
KO of *Runx3* inhibits CD43 core 2 O-glycosylation but not the expression of CD43 and several activation markers and adhesion molecules on mLN CD8^+^ T cells during H1N1 IAV infection. Age- and sex-matched littermate control (Con) and *Runx3* general KO (KO) mice were infected intranasally with 1 LD_50_ of H1N1 PR8 strain. After 6 and 9 days, the cells from mLNs were subjected to flow cytometry analysis. (**A**,**B**) Suppression of CD43 core 2 O-glycosylation but not its expression on mLN CD8^+^ T cells by *Runx3* KO during IAV infection. CD43 core 2 O-glycans (Glyco-CD43) were assessed by the 1B11 glycosylated-CD43 monoclonal antibody (**A**) and CD43 protein level was determined by S11 monoclonal antibody against non-glycosylated form of CD43 (**B**) on CD3^+^CD8^+^-gated cells. Representative FACS histogram plots evaluated on day 6 (d6) and/or d9 pi are shown in the panels, and the mean fluorescence intensity (MFI) is shown in the bar graphs. Results are presented as means ± SE (n = 4–5 in panel (**A**), n = 3 in panel (**B**)). (**C**–**F**) *Runx3* KO has a minor effect on the expression of CD44 (**C**), CD69 (**D**), CD11a (**E**), and CD54 (**F**) on mLN CD8^+^ T cells. Representative FACS histogram plots evaluated on d6 and/or d9 pi are shown in the panels, and MFI is shown in the bar graphs. Results are presented as means ± SE (n = 3–5). An unpaired Student’s *t* test was employed for comparison in (**A**–**F**). ** *p* < 0.01; *** *p* < 0.001 vs. control mice. ns, not significant.

**Figure 3:**
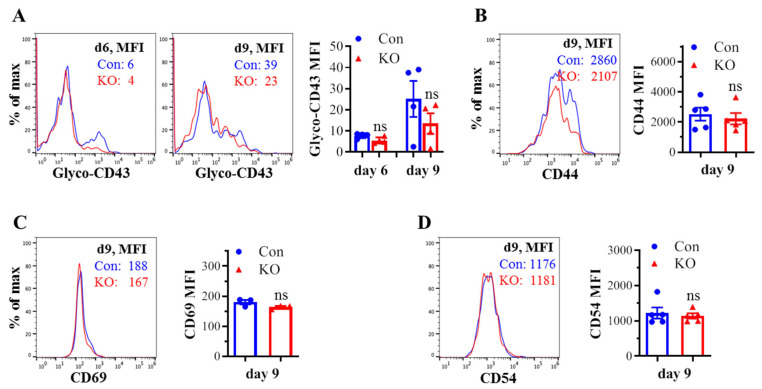
Effects of *Runx3* KO on mLN CD4^+^ T cell phenotypes following H1N1 IAV infection. Age- and sex-matched littermate control (Con) and *Runx3* general KO (KO) mice were infected intranasally with 1 LD_50_ of H1N1 PR8 strain, and the cells from mLNs on day 6 (d6) and d9 pi were subjected to flow cytometry analysis. (**A**) *Runx3* KO has a modest effect on CD43 core 2 O-glycosylation on mLN CD4^+^ T cells following IAV infection. CD43 core 2 O-glycans (Glyco-CD43) were assessed by the 1B11 glycosylated-CD43 monoclonal antibody on CD3^+^CD4^+^-gated cells. Representative FACS histogram plots evaluated on d6 and/or d9 pi are shown in the panels, and the mean fluorescence intensity (MFI) is shown in the bar graph. Results are presented as means ± SE (n = 4). (**B**–**D**) The expression of CD44 (**B**), CD69 (**C**), and CD54 (**D**) on CD3^+^CD4^+^-gated cells was barely affected by *Runx3* KO. Representative FACS histogram plots evaluated on d9 pi are shown in the panels, and MFI is shown in the bar graphs. Results are presented as means ± SE (n = 3–5). An unpaired Student’s *t* test was employed for comparison in (**A**–**D**). ns, not significant.

We assessed the effect of *Runx3* KO on CD8^+^ T cell phenotypes including CD43 glycosylation and cell surface expression of activation markers and cell adhesion molecules in mLNs following H1N1 IAV infection. The tool to analyze O-linked glycosylation is limited. However, some antibodies that specifically recognize protein glycosylation patterns can be utilized for glycan analysis. The monoclonal antibody 1B11 binds the E-selectin ligand CD43 only when modified with core 2 O-glycosylation [34] and hence, has been widely used to assess CD43 core 2 O-linked glycosylation on activated CD8^+^ T cells [19,20,21,22]. By using the 1B11 glycosylated-CD43 antibody, we found that the synthesis of CD43 core 2 O-glycans on mLN CD8^+^ T cells was inhibited ~83% by *Runx3* KO on days 6 and 9 post IAV infection (Figure 2A). The inhibition of CD43 core 2 O-glycosylation was not due to the suppression of CD43 expression, as *Runx3* KO had a minor effect on CD43 protein levels detected with a monoclonal antibody (S11) against non-glycosylated form of CD43 (Figure 2B). Moreover, we found that *Runx3* KO modestly enhanced mLN CD8^+^ T cell surface expression of CD44 [35], which is a marker of activated T cells during IAV infection (Figure 2C). CD69 [36], a transient activation marker of CD8^+^ T cells, was detected to be low on mLN CD8^+^ T cells, and its expression was minimally affected by *Runx3* KO during IAV infection (Figure 2D). In addition, *Runx3* KO had a minor effect on the expression of cell adhesion molecules CD11a and CD54 on mLN CD8^+^ T cell during IAV infection (Figure 2E,F). In contrast to mLN CD8^+^ T cells, the level of CD43 core 2 O-glycosylation on mLN CD4^+^ T cells was relatively low and not significantly altered by *Runx3* KO during IAV infection (Figure 3A). The expression levels of CD44, CD69, or CD54 on mLN CD4^+^ T cells were barely affected by *Runx3* KO following IAV infection (Figure 3B–D).

**Figure 4:**
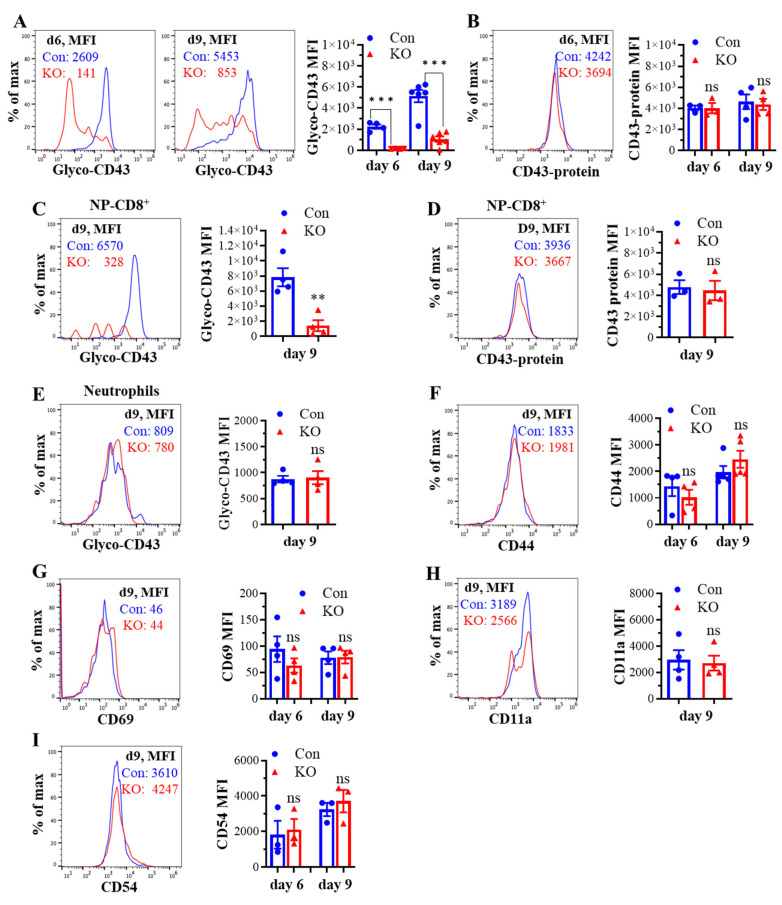
KO of *Runx3* markedly inhibits CD43 core 2 O-glycosylation but not the expression of CD43 and several activation markers and adhesion molecules on lung CD8^+^ CTLs during H1N1 IAV infection. Age- and sex-matched littermate control (Con) and *Runx3* general KO (KO) mice were infected intranasally with 1 LD_50_ of H1N1 PR8 strain, and the cells from BALFs and whole lungs on day 6 (d6) and d9 pi were subjected to flow cytometry analysis. (**A**–**D**) A marked inhibition of CD43 core 2 O-glycosylation but not its expression on total and NP_366–374_-specific lung CD8^+^ CTLs by *Runx3* KO. CD43 core 2 O-glycans (Glyco-CD43) and protein levels were assessed on CD3^+^CD8^+^-gated cells from BALFs (**A**,**B**) and NP_366–374_-specific CD8^+^ CTLs (NP-CD8^+^) from whole lungs of IAV-infected mice (**C**,**D**). Representative FACS histogram plots evaluated on d6 and/or d9 pi are shown in the panels, and the mean fluorescence intensity (MFI) is shown in the bar graphs. Results are presented as means ± SE. In panel (**A**), n = 4–6. In panel (**B**), n = 3–4. In panel (**C**), n = 4. In panel (**D**), n = 3. (**E**) *Runx3* KO has a minor effect on CD43 core 2 O-glycosylation on BLAF neutrophils (CD11b^+^Ly6G^high^). Representative FACS histogram plot evaluated on d9 pi is shown in the panel. MFI is shown in the bar graphs, and the results are presented as means ± SE (n = 4). (**F**–**I**) The expression of CD44 (**F**), CD69 (**G**), CD11a (**H**), and CD54 (**I**) on BALF CD8^+^ CTLs (CD3^+^CD8^+^) was barely affected by *Runx3* KO. Representative FACS histogram plots evaluated on d9 pi are shown in the panels, and MFI is shown in the bar graphs. Results are presented as means ± SE. In panel (**F**–**H**), n = 4. In panel (**I**), n = 3. An unpaired Student’s *t* test was employed for comparison in (**A**–**I**). ** *p* < 0.01; *** *p* < 0.001 vs. control mice. ns, not significant.

We next determined the effect of *Runx3* KO on lung CD8^+^ T cell phenotypes following H1N1 IAV infection. As shown in Figure 4A, the IAV-induced synthesis of CD43 core 2 O-glycans on lung CD8^+^ effector T cells from control mice was much higher than that of mLN CD8^+^ T cells (Figure 2A) and was inhibited ~85% by *Runx3* KO. The marked inhibition of CD43 core 2 O-glycosylation on lung CD8^+^ T cells correlates with a huge reduction in the numbers of pulmonary CD8^+^ CTLs during IAV infection as we showed recently [32]. We further found that the IAV-induced synthesis of CD43 core 2 O-glycans on IAV nucleoprotein (NP)_366–374_-specific CD8^+^ effector T cells was profoundly suppressed by *Runx3* KO (Figure 4C), which correlates with ~90% reduction in NP_366–374_-specific CD8^+^ effector T cell populations in IAV-infected mouse lungs as we reported recently [32]. The inhibition of CD43 core 2 O-glycosylation was not due to the reduction in CD43 expression, since *Runx3* KO had a minor effect on CD43 protein levels in lung total and NP_366–374_-specific CD8^+^ effector T cells detected with a monoclonal antibody (S11) against non-glycosylated form of CD43 (Figure 4B,D). In contrast, CD43 core 2 O-glycosylation on lung neutrophils was barely affected by *Runx3* KO following IAV infection (Figure 4E). Consistent with the findings of mLN CD8^+^ T cells, lung CD8^+^ T cell surface expression of CD44, CD69, CD11a, and CD54 was also not significantly altered by *Runx3* KO during IAV infection (Figure 4F–I). These findings indicate that Runx3 is a crucial mediator of core 2 O-linked glycosylation of selectin ligand CD43 on activated CD8^+^ T cells in response to H1N1 IAV infection.

### 2.3. Runx3 KO Minimally Affects Lung CD8^+^ T Cell Death in Response to H1N1 IAV Infection

**Figure 5:**
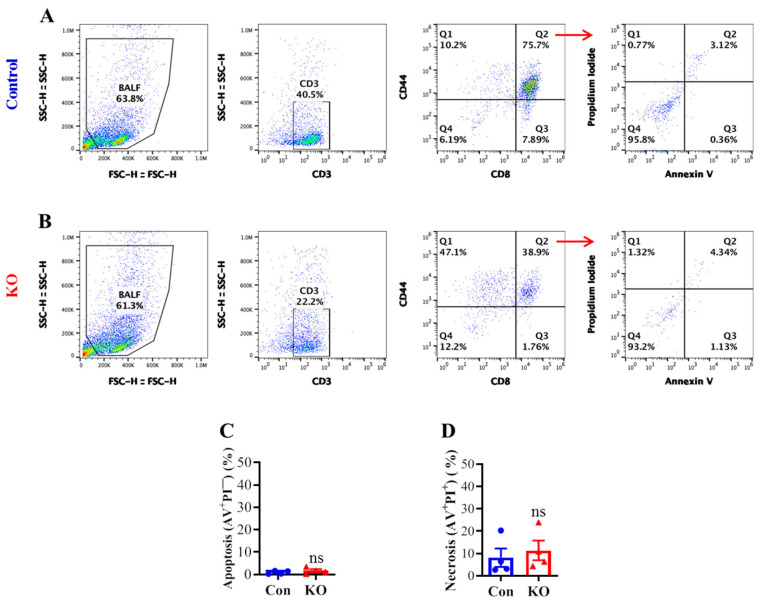
A general *Runx3* KO in adult mice minimally affects lung CD8^+^ CTL cell death in response to H1N1 IAV Infection. Age- and sex-matched littermate control (Con) and *Runx3* general KO (KO) mice were infected intranasally with 1 LD_50_ of H1N1 PR8 strain, and BALFs on day 9 pi were collected and subjected to flow cytometry analysis. Activated CD8^+^ T cells (CD8^+^CD44^+^) were identified from the CD3^+^-gated cell populations and subjected to annexin V (AV) and propidium iodide (PI) cell death assay as shown in the representative FACS plots (**A**,**B**). Lung CD8^+^ effector T cell apoptosis ((**C**), AV^+^PI^−^) and necrosis ((**D**), AV^+^PI^+^) are shown in the bar graphs. Results are presented as means ± SE (n = 4). An unpaired Student’s t test was employed for comparison in (**C**,**D**). ns, not statistically significant.

To determine if the marked reduction in lung CD8^+^ T cell CD43 core 2 O-glycosylation and populations by *Runx3* KO correlates with an enhanced cell death, we performed flow cytometry with annexin V (AV) and propidium iodide (PI) cell death assay. Flow cytometry analyses of BALF activated CD8^+^ T cells (CD3^+^CD8^+^CD44^+^) revealed that *Runx3* KO did not significantly alter the cell apoptosis (AV^+^PI^−^) and necrosis (AV^+^PI^+^) following IAV infection (Figure 5A–D). These results indicate that a general *Runx3* deficiency in adult mice minimally affects lung effector CD8^+^ T cell death by H1N1 IAV infection.

### 2.4. Runx3 KO Reduces Core 2 O-Glycosylation of CD43 on CD8^+^ T Cells Activated In Vitro

**Figure 6:**
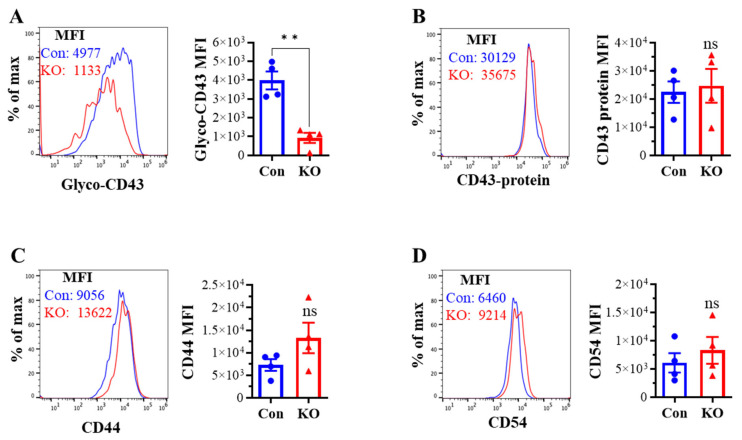
KO of *Runx3* reduces core 2 O-glycosylation of CD43 on activated CD8^+^ T Cells in vitro. Spleen CD8^+^ T cells were isolated from naïve (untreated) control (Con) or *Runx3* KO (KO) mice by negative selection, treated with anti-CD3ε (2 µg/mL) and anti-CD28 (2 µg/mL) antibodies for activation and then cultured with recombinant mouse IL-2 (100 U/mL) for 5 days. The activated CD8^+^ T cells were then subjected to flow cytometry analysis to assess core 2 O-glycan synthesis of CD43 ((**A**), Glyco-CD43), the cell surface expression of CD43 (**B**), CD44 (**C**), and CD54 (**D**). Representative FACS histogram plots are shown in the panels, and MFI is shown in the bar graphs. Results are presented as means ± SE (n = 4). An unpaired Student’s *t* test was employed for comparison in (**A**–**D**). ** *p* < 0.01 vs. control mice. ns, not significant.

To determine if the reduction in CD43 core 2 O-glycosylation by *Runx3* KO is an intrinsic effect on activated CD8^+^ T cells, spleen CD8^+^ T cells were isolated from naive (untreated) littermate control or *Runx3* KO mice by negative selection, treated with anti-CD3ε and anti-CD28 antibodies for activation, and then cultured in completed RPMI-1640 medium supplemented with recombinant mouse IL-2 for 5 days. Flow cytometry analysis revealed that CD43 core 2 O-glycan synthesis, but not its protein level, was readily inhibited by *Runx3* KO on the CD8^+^ T cells activated in vitro (Figure 6A,B). Moreover, we found that CD44 and CD54 were modestly upregulated by *Runx3* KO on the activated CD8^+^ T cells. These findings indicate that *Runx3* deficiency causes an intrinsic defect of CD43 core 2 O-glycosylation on activated CD8^+^ T cells.

## 3. Discussion

We recently reported that Runx3 is required for pulmonary generation of CD8^+^ CTLs against IAV infection [32]. To unravel the potential mechanisms underlying Runx3 regulation of pulmonary CD8^+^ CTLs due to IAV infection, the effects of *Runx3* deficiency on CD8^+^ T cell local expansion and phenotypes were investigated in this study using an inducible global *Runx3* KO mouse model. Our findings indicate that Runx3 is required for core 2 O-glycosylation of selectin ligand CD43 on activated CD8^+^ T cells and differently regulates CD8^+^ T cells expansion in mLNs and lungs in response to H1N1 IAV infection.

By using a mature T cell-specific *Runx3* KO mouse model, it was shown that *Runx3* KO impairs CD8^+^ effector T cell expansion and function in spleens against lymphocytic choriomeningitis virus (LCMV) infection that causes a systemic infectious disease [37]. Mechanistically, Runx3 guards the generation of splenic CD8^+^ CTLs against deviation towards follicular helper T (Tfh) cell lineage [37]. Runx3 is also required for memory CD8^+^ CTL formation and the differentiation and maintenance of tissue-resident memory CD8^+^ T cells (T_RM_) in non-lymphoid tissues in response to LCMV infection [38,39]. However, less is known as to how Runx3 mediates CTLs in non-lymphoid tissues, especially in the context of respiratory viral infection. Here, we provided substantial evidence that a general *Runx3* KO in adult mice promoted mLN enlargement and expansion of CD8^+^ and CD4^+^ T cells during H1N1 IAV infection, whereas *Runx3* KO greatly suppressed pulmonary generation of CD8^+^ CTLs during IAV infection as we showed recently [32]. This indicates that Runx3 differently regulates CD8^+^ T cells expansion in mLNs and lungs following IAV infection. We further showed that *Runx3* deficiency minimally affected lung effector CD8^+^ T cell death in response to H1N1 IAV infection. These data suggest that the defects in *Runx3^−^*^/*−*^ CD8^+^ T cell egression from mLNs, trafficking or homing to lungs, or local lung expansion may be involved in the reduced formation of pulmonary CD8^+^ CTLs against IAV infection.

It has been demonstrated that core 2 O-linked glycosylation of CD43 and PSGL-1 is required for the generation of functional selectin ligands that mediate the homing of activated CD8^+^ T cells to non-lymphoid tissues including the lungs in vivo [14,15,16,17,18,19,20,21,22]. In control mice, we found that the IAV-induced synthesis of CD43 core 2 O-glycans on lung CD8^+^ effector T cells was much higher than that of mLN CD8^+^ T cells, suggesting that the activated CD8^+^ T cells may undergo continuous core 2 O-linked glycosylation (differentiation) during trafficking to IAV-infected lungs. Interestingly, we found that *Runx3* KO markedly inhibited CD43 core 2 O-glycosylation on activated CD8^+^ T cells from IAV-infected mLNs and lungs but minimally affected the cell surface expression of CD43, activation markers (CD44 and CD69), and cell adhesion molecules (CD11a and CD54). It should be noted that CD43 core 2 O-glycosylation on the isolated CD8^+^ T cells activated in vitro was also inhibited by *Runx3* KO. This indicates that *Runx3* deficiency causes an intrinsic defect of CD43 core 2 O-glycosylation on activated CD8^+^ T cells. In contrast to CD8^+^ T cells, *Runx3* KO had a modest effect on the levels of CD43 core 2 O-glycosylation on mLN CD4^+^ T cells and lung neutrophils following H1N1 IAV infection, indicating a specific role of Runx3 in CD8^+^ T cell differentiation. The synthesis of core 2 O-glycans is mediated by the actions of a number of glycosyltransferases [14,15,20,22]. The polypeptide GalNAc transferase 3 (*Galnt3*) transfers an N-acetylgalactosamine (GalNAc) to the hydroxyl group of a serine or threonine residue to initiate O-linked oligosaccharide biosynthesis. This is then followed by core 2 β1,6 *N*-acetylglucosaminyl-transferase-I [C2GlcNAcT-I (*Gcnt1*)] to mediate the synthesis of core 2 O-glycans on activated CD8^+^ T cells [19]. There are 3 C2GlcNAcT isoforms that are transcribed from *Gcnt1*, *Gcnt3*, and *Gcnt4*, respectively [40]. Only *Gcnt1* expression was induced in activated CD8^+^ T cells by LCMV infection [19], and KO of *Gcnt1* markedly reduced CD43 O-glycan synthesis on activated CD8^+^ T cells and the cell binding to P- and E-selectins as well as memory CD8^+^ T cell trafficking into the skin [20]. Besides *Gcnt1*, other glycosyltransferases such as *B4galt5*, *B3gnt3*, *Fut7*, and *St3gal4* that extend lactosamine repeats, fucose and terminal sialic acid, respectively, are also involved in core 2 O-glycan formation [15,20]. By analyzing the previous RNA sequencing data from LCMV-infected mouse spleen *Runx3^−^*^/*−*^ CD8^+^ effector T cells (GEO accession # GSE81888) [37], we found that the expression levels of *Galnt3* and *Gcnt1* were inhibited 75% or 89%, respectively, by *Runx3* deficiency compared with control CD8^+^ effector T cells, whereas the expression levels of *B4galt5*, *B3gnt3*, *Fut7*, and *St3gal4* were not significantly altered by *Runx3* KO. Moreover, we analyzed previous microarray data and found that *Galnt3* and *Gcnt1* were also downregulated by *Runx3* KO in germline-targeted *Runx3^−^*^/*−*^ CD8^+^ T cells activated in vitro (GEO accession # GSE50131) [41]. Hence, the marked downregulation of *Galnt3* and *Gcnt1* by *Runx3* deficiency observed in activated CD8^+^ effector T cells highly supports our findings that Runx3 is a crucial mediator of core 2 O-glycosylation of CD43 on activated CD8^+^ T cells in response to IAV infection. Collectively, we infer that the defective core 2 O-glycosylation of selectin ligands by *Runx3* KO may impair the activated CD8^+^ T cell trafficking to inflamed lungs to combat IAV infection. This hypothesis merits further investigation by using an adoptive transfer approach.

The *Gcnt1*-dependent CD43 core 2 O-glycosylation has recently been identified as a sensitive marker of Notch signaling in activated CD8^+^ and CD4^+^ T Cells [23]. Notch signaling is critical for T cell activation, differentiation, and effector function [24], and the impaired Notch signals in T cells reduce their protective functions against bacterial, viral, fungal, and parasitic infections in mice [25,26,27,28]. Activation of Notch receptors triggers nuclear translocation of the Notch intracellular domain to mediate the expression of Notch target genes. As both Notch signaling and Runx3 are required for the *Gcnt1*-dependent core 2 O-glycosylation of CD43 on activated CD8^+^ T cells, our findings suggest that Runx3 may be a Notch target gene or cooperate with Notch signaling to mediate core 2 O-glycosylation of CD43 by regulating the expression of *Gcnt1* and/or *Galnt3* in activated CD8^+^ T cells. Indeed, it has been shown that Runx3 is a direct Notch target gene in human endothelial cells [42]. Although both Notch signals and Runx3 are directly involved in CD8^+^ T cell activation, proliferation, and differentiation [24,37,43], we found that a general *Runx3* KO in adult mice markedly promoted mLN enlargement and expansion of CD8^+^ and CD4^+^ T cells during H1N1 IAV infection. This suggests that the Runx3-driven signals in other types of cells also critically regulate CD8^+^ and CD4^+^ T cell activation and expansion in mLNs. We recently showed that Runx3 was strongly expressed in CCR2^+^ immune cells in mouse lungs by IAV infection and was induced in activated DCs and macrophages [32]. Among murine DCs, Runx3 expression is mainly restricted to conventional DC subset-2 (cDC2) [44,45]. Lung cDC1 cells predominantly elicit cytotoxic CD8^+^ and T helper type 1 (Th1) CD4^+^ T cell responses, while cDC2 provokes Th2, regulatory T cell (Treg), and Tfh immune responses [13,46,47,48,49,50]. Th2 response inhibits antiviral immunity and delays viral clearance [51,52,53,54], while Treg cells dampen CD4^+^ and CD8^+^ T cell proliferation and inflammatory signals, preventing immune responses [55,56]. Recent studies have shown that CD11c-specific *Runx3* KO inhibits more than 80% Th2 response to ovalbumin in spleens and causes a substantial reduction in Foxp3^+^ Treg cells in colon lamina propria under the steady state [44,45]. Hence, we posit that *Runx3* deficiency in cDC2 would suppress Th2 and Treg responses and result in a host immune balance skewing to Th1 immunity, which promotes mLN local expansion of CD8^+^ and CD4^+^ T cells during IAV infection in the *Runx3* KO mice. It would be interesting to test this hypothesis using DC-specific *Runx3* KO models.

In conclusion, we provide evidence showing that Runx3 differently regulates CD8^+^ T cells expansion in mLNs and lungs in response to H1N1 IAV infection. We further show that Runx3 is required for core 2 O-glycosylation of selectin ligand CD43 on activated CD8^+^ T cells by regulating the expression of *Gcnt1* and/or *Galnt3*.

## 4. Materials and Methods

### 4.1. Inducible Runx3 Global KO Mouse Model

All animal experiments in the present study were approved by the Institutional Animal Care and Use Committee at the University of Texas Health Science Center at Tyler. We utilized Cre/Lox technology to generate an inducible *Runx3* global KO mouse model as we described recently [32]. *Runx3^f/f^* mice (B6.129P2-*Runx3^tm1Itan^*/J, #008773) in which the exon 4 was flanked by loxP sites, and *CreER^T2^-ROSA26* mice (B6.129-*Gt(ROSA)26Sor^tm1(cre/ERT2)Tyj^*/J, #008463) that allow for tamoxifen-induced temporal control of floxed gene expression were obtained from Jackson Laboratory (Bar Harbor, ME, USA). The *Runx3^f/f^* mice were crossed with *CreER^T2^-ROSA26* mice for five generations, and the desirable genotype of inducible *Runx3* KO mice were successfully obtained by genotyping according to the previous protocols [57,58]. To induce a general KO of *Runx3*, 6- to 8-week-old inducible *Runx3* KO (*Runx3^f/f^:ROSA26-ERCre^+^*) and littermate control (*Runx3^f/f^:ROSA26-ERCre^-^*) mice were given tamoxifen (150 mg/kg body weight) by intraperitoneal (i.p.) injection every other day for ten days as described previously [59]. The KO efficiency was verified by PCR and Western blot analyses two weeks after tamoxifen treatment as we showed recently [32]. We used age- and sex-matched *Runx3* KO and littermate control mice one month post tamoxifen treatment for the present study.

### 4.2. H1N1 Infection and Animal Experiments

Age- and sex-matched littermate control and *Runx3* general KO mice on C57BL/6 genetic background were anesthetized by i.p. injection of ketamine (100 mg/kg) and xylazine (8.5 mg/kg), and then given intranasally with 35 μL saline containing 1 LD_50_ (~360 plaque-forming units (pfu)) H1N1 PR/8/34 strain or 35 μL saline alone as experimental controls. H1N1 PR/8/34 strain was from Charles River (Wilmington, MA, USA). These experiments were performed under a class II biosafety cabinet. Disposable PPE, such as gowns, gloves, masks, and shoe covers were used to prevent infection of personnel. Working areas exposed to live virus were disinfected with 70% ethanol, and hands were washed after experiments. The IAV-infected mice were housed in a designated BSL2 room and observed daily for signs of distress, body weight loss, and animal survival. Mice having a loss of more than 30% of their initial body weight were humanely euthanized and counted as dead. Bronchoalveloar lavage fluids (BALFs), lung, and mLN tissues were collected on days 3, 6, and 9 pi as we described [32].

### 4.3. Preparation of Lung and mLN Single-Cell Suspension

The preparation of lung single-cell suspension was performed as we described recently [32]. For the preparation of mLN single-cell suspension, the mLNs from IAV-infected mice were homogenized in Dounce glass tissue grinders with 2 mL of RPMI-1640 medium containing 2.5% fetal bovine serum (FBS). The mLN cell suspensions were then passed through 100 and 70 µm nylon strainers. After lysis of red blood cells, mLN cell pellets were resuspended in RPMI-1640 medium supplemented with 2.5% FBS for cell counting. These experiments were performed under a class II biosafety cabinet with PPE (gloves, masks, and gowns or lab coat) protection. Working areas exposed to live virus were disinfected with 70% ethanol, and hands were washed after experiments.

### 4.4. Flow Cytometry Analysis

BALF cells, mLN, or lung single cell suspensions were resuspended in cell staining buffer (no. 420201, BioLegend, San Diego, CA, USA) and blocked with TruStain FcX (anti-mouse CD16/32) antibody (no. 101320, BioLegend, San Diego, CA, USA) for 10–15 min on ice. The cells were then incubated 30 min with the following specific fluorochrome-conjugated antibodies or isotype controls (BioLegend, San Diego, CA, USA ) in the dark at 4 °C: Alexa Fluor-488 anti-CD45, PE anti-CD3, APC anti-CD4, FITC anti-CD4, PE-Cy7 anti-CD8α, APC anti-glycosylated CD43 (1B11), FITC anti-CD43, FITC anti-CD44, APC anti-CD11a, PE/Cy5 anti-CD69, Pacific Blue anti-CD54, APC anti-CD11b, PE anti-Ly6G, or FITC isotype control antibodies. PE-conjugated tetramer specific for H-2Db IAV NP_366–374_ (ASNENMETM) was from MBL International Corporation (Woburn, MA, USA). After washing, stained cells were acquired in an Attune NxT flow cytometer (Thermo Fisher Scientific, Waltham, MA USA). Cell death was determined by flow cytometry using APC annexin V apoptosis detection kit with propidium iodide (no. 640932, BioLegend, San Diego, CA, USA). Flow cytometry data were analyzed by using FlowJo software v10.6.2.

### 4.5. Western Blot Analysis

Western blot analysis was performed essentially as we described previously [60]. A validated Runx3 antibody (no. 9647) from Cell Signaling Technology (Danvers, MA, USA) was employed for the analysis.

### 4.6. CD8^+^ T Cell Isolation and Activation

Spleen CD8^+^ T cells were isolated from naive (untreated) littermate control or *Runx3* KO mice by a negative selection approach using CD8α^+^ T cell isolation kits (no. 130-104-075, Miltenyi Biotec, Auburn, CA, USA). Spleen single-cell suspension was prepared in Dounce glass tissue grinders with 4 mL of RPMI-1640 medium containing 2.5% FBS and passed through 100 µm nylon strainer. After lysis of red blood cells, spleen cells were resuspended in MACS buffer containing 1x PBS, 0.5% bovine serum albumin (BSA), and 2 mM EDTA. CD8^+^ T cells were subsequently isolated by negative selection using antibody-conjugated magnetic microbeads and MACS LS columns according to the manufacturer’s protocol (Miltenyi Biotec, Auburn, CA, USA). For cell stimulation, purified CD8^+^ T cells were seeded at 1 × 10^6^ cells/well in 12-well plates precoated with anti-CD3ε (2 µg/mL, no. 100302, BioLegend) and anti-CD28 (2 µg/mL, no. 102102, BioLegend) antibodies and cultured in RPMI-1640 medium supplemented with 10% FBS and 50 μg/mL penicillin/streptomycin as described [43]. After 48 h, CD8^+^ T cells were transferred to new and uncoated wells at 5 × 10^5^ cells/well in completed RPMI-1640 medium supplemented with 100 U/mL recombinant mouse IL-2 (no. 575402, BioLegend) and cultured for 5 days. Cells were counted every 24 h and readjusted to 5 × 10^5^ cells/well with fresh media containing 100 U/mL IL-2.

### 4.7. Statistical Analysis

Statistical calculations were performed by using GraphPad Prism 9 (GraphPad Software, La Jolla, CA, USA). Data are expressed as mean ± SE. An unpaired Student’s *t* test was employed for comparison between means of two groups. Two-way ANOVA, followed by Bonferroni’s correction, was employed to compare a response with two factors. Values were considered statistically significant at *p* < 0.05.

## Data Availability

All datasets generated for this study are included in the article.
